# Mitigating CO_2_ emissions associated with digital economy sectors through whole supply chain management

**DOI:** 10.1371/journal.pone.0323350

**Published:** 2025-05-20

**Authors:** Wenhuan Wang, Zijian Cai, Yongzhen Zhu, Dian Yu, Jingjing Zhan, Xiaoqi Li, Xiaoyu Wang

**Affiliations:** 1 School of Public Administration, Zhejiang University of Technology, Hangzhou, China; 2 Zhejiang Institution of Talent Development, Hangzhou, China; 3 School of Sociology, Central China Normal University, Wuhan, China; 4 Graduate School of Education, Beijing Foreign Studies University, Beijing, China; 5 New Technology Center, Ministry of Science & Technology, Beijing, China; 6 Inner Mongolia University of Finance and Economics, Hohhot, China; Guilin University of Aerospace Technology, CHINA

## Abstract

As China’s digital economy sectors rapidly expand, the growing demand for coal-based electricity has become a significant source of CO_2_ emissions. However, the mechanism driving these emissions within supply chains remain unclear, hindering targeted carbon management. This study addresses this gap by providing a comprehensive analysis of CO_2_ emissions thorough the whole supply chain perspective, covering income-, production-, betweenness-, and consumption-based perspectives, along with upstream and downstream supply chain paths. It employs Leontief and Ghosh input-output (IO) frameworks and structural path analysis. The results indicate: (1) The core industry sector of the digital economy (CIDE) ranks highest in CO_2_ emissions from consumption-based perspective, while the industrial digitalization sector (IDS) ranks highest from both consumption- and betweenness-based perspectives. (2) Inter provincial flows are the main source driving the digital economy sectors’ supply chain embodied CO_2_ emissions from consumption-based perspective, while labor compensation is the primary source driving its enabled CO_2_ emissions from income-based perspective. (3) High-carbon upstream and downstream supply chain paths driven by the digital economy sectors are short, with the power and heat production and supply sector and IDS playing crucial roles within these chains. Based on these findings, policy recommendations are provided to optimize supply chain structures, promote green consumption, and integrate carbon management into sector-specific strategies to reduce emissions across both upstream and downstream paths.

## 1. Introduction

As a key engine of economic growth [1], the digital economy has permeated production activities across various industries [2,3]. In 2023, China’s digital economy accounted for 42.8% of its GDP, ranking second globally [4]. It has also made positive contributions to China’s CO_2_ reduction targets [5], primarily by facilitating energy transitions [6], industrial transitions [7], improving green economic efficiency [8], and alleviating energy poverty [9]. However, the advancement of digital industrialization has intensified dependence on coal [10] and electricity [11]. It has increased material resource consumption and energy efficiency [12], thereby exacerbating the CO_2_ emissions.

Emissions within the supply chains of digital economy sectors are on the rise [13]. While direct carbon emissions of industries typically account for only 14% of total supply chain emissions [14], CO_2_ emissions across these supply chains are often significantly overlooked. This indicates that meeting low-carbon targets across the supply chains through the sectoral management of emission reductions alone is challenging [15]. Consequently, it necessitates collaborative efforts to mitigate CO_2_ emissions related to the digital economy sectors through whole supply chain management.

**Fig 1:**
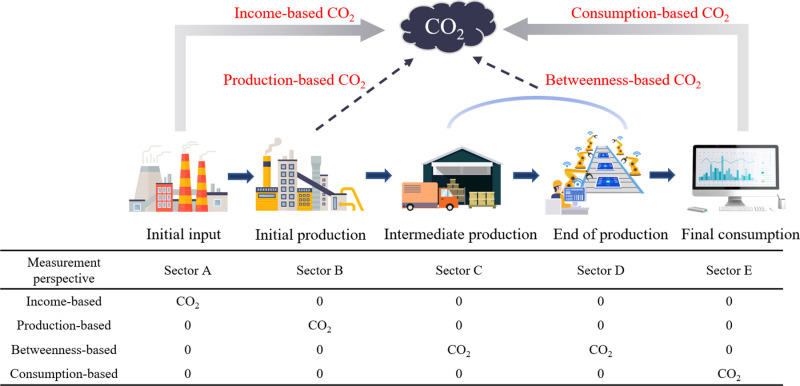
A schematic diagram illustrating four perspectives for estimating CO_2_ emissions.

CO_2_ emissions generated by economic consumption activities from different perspectives of the supply chains correspond to varied policy implications [16,17]. Income-based accounting serves to regulate human capital investment behaviors, production-based accounting aims to manage emission efficiencies [18], betweenness-based accounting supports policy measures to improve production efficiency [19], and consumption-based accounting guides optimization of consumption behaviors [20]. Fig 1 illustrates an example of a whole supply chain consisting of five sectors. Within this chain, CO_2_ emissions are allocated to the initial input sector, the direct emitting production sector, the emission transmitting production sector, and the final consuming sector, based on income, production, betweenness, and consumption, respectively. These allocations reflect CO_2_ emissions driven by initial inputs, direct production, intermediation production, and final consumption.

Existing research has examined the measurement and analysis of CO_2_ emissions in supply chains of the digital economy sectors examined from diverse perspectives. Scholars initially focused on CO_2_ emissions issues of information and communication technology (ICT) sector. For example, Belkhir and Elmeligi [21] assessed the global emissions footprint of ICT from production-based perspective. Wang et al. [22] assessed the embodied CO_2_ emissions from a consumption-based perspective and explored drivers in the ICT sectors. Zhou et al. [23] explored production and consumption-based CO_2_ emissions, drivers and supply chains of China’s ICT sectors. Yuan et al. [24] analyzed the year-by-year changes and impacts of enabled emissions from an income-based perspective in the ICT industry using a subsystem input-output model. While ICT is an important part of the core industry sector of the digital economy (CIDE) [25], it is not entirely equivalent to the digital economy. As the concept of the digital economy continues to evolve, scholars are increasingly focusing on the CO_2_ emissions issues within the digital economy sectors. Based on ICT research, Zhou et al. [26] included the information and communication services sectors and analyzed the carbon impacts of embodied and enabled CO_2_ emissions of China’s ICT sectors from consumption and income-based perspective. Additionally, Wang et al. [27] defined the digital economy sectors according to National Bureau of Statistics of China and explored the betweenness-based CO_2_ emissions from China’s digital economy sectors.

However, these studies typically focus on one to three perspective-based estimation methods, revealing a gap in comprehensive research that encompasses the whole supply chain perspectives of the digital economy sectors. Moreover, the contributions of final demand and initial inputs to CO₂ emissions in the supply chains of the digital economy sectors remain unclear. Additionally, the concept of the digital economy supply chain encompasses both upstream and downstream components, yet previous studies have failed to analyze the path characteristics of CO_2_ emissions within these complex supply chain networks.

Research gaps hinder understanding of CO_2_ emissions in digital economy supply chains, undermining reduction policies and slowing decarbonization. Consequently, this study aims to enhance understanding and mitigate emissions through whole supply chain management by answering the following three questions: (1) How to identify CO_2_ emissions of digital economy sectors from different perspective? (2) How do final demand and initial inputs drive CO_2_ emissions in digital economy supply chains? (3) What are the comprehensive characteristics of CO_2_ emissions across upstream and downstream supply chain paths?

Many environmental studies narrow their focus to the provincial level [28] or compare provinces within a country [29] to more thoroughly analyze regional specificities. This study selects Zhejiang Province in China as a case study, considering its unique characteristics for two primary reasons. Firstly, the province’s digital economy contributes 48.6% to its GDP [30], making it a highly representative exemplar of China’s digital economy forefront and future trajectory. Secondly, the embodied carbon emissions of China’s digital economy cannot be overlooked (accounting for 6.10% of the total in 2017) [13]. However, Zhejiang Province exhibits significant negative carbon emission spillover effects [31], making it an excellent case study for analyzing how to further reduce CO_2_ emissions associated with the digital economy and expand its green benefits.

**Fig 2:**
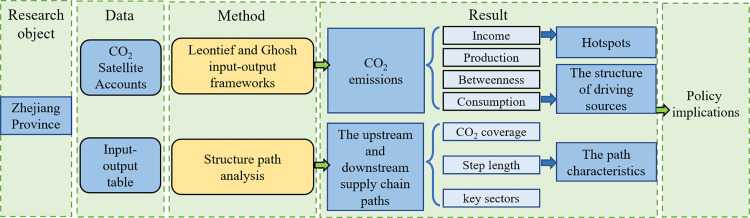
Research framework of this research.

Using an environmental input-output model and CO₂ satellite accounts, this study examines CO₂ emissions from the digital economy sectors from income-, production-, betweenness- and consumption-based perspectives of the supply chain. It also investigates the structures of driving sources of embodied and enabled CO_2_ emission. Moreover, delving deep into the primary driving sources, this study uses structural path analysis (SPA) to elucidate the characteristics of CO_2_ emissions driven by the digital economy sectors across its upstream and downstream supply chain paths. Fig 2 shows the research framework of this article.

Thus, this study makes several key contributions: (1) It conducts a comprehensive investigation into the CO_2_ emission characteristics of the digital economy sectors’ supply chains, focusing on income-, production-, betweenness-, and consumption-based perspectives. (2) It provides an in-depth analysis of the types of final demand and initial inputs that primarily drive embodied and enabled CO_2_ emissions in the digital economy sectors’ supply chains, as represented in the input-output table (IOT). (3) It employs Leontief and Ghosh models within structural path analysis to trace CO_2_ emissions through the upstream and downstream supply chain paths of the digital economy sectors, clarifying the features of high-carbon supply chain paths such as CO_2_ coverage, path length, and terminal sectors. The findings of this study inform the development of comprehensive CO_2_ reduction policies for Zhejiang’s digital economy, enhancing environmental benefits through whole supply chain management. Additionally, these findings help promote CO_2_ reduction initiatives of China’s digital economy, significantly contributing to the nation’s achievement of its CO_2_ reduction goals. Furthermore, given the digital economy’s profound impact on the global economy, this study provides valuable insights for other countries and regions to understand their environmental impacts and manage CO_2_ emissions, thereby fostering a global consensus and encouraging collaborative CO_2_ reduction policies.

The remainder of this study is organized as follows: Section 2 describes the methods and data of this study. Section 3 summarizes the results. Section 4 discusses these findings. Finally, Section 5 summarizes the conclusions and policy implications.

## 2. Methods and data

### 2.1. Four perspectives for estimating CO_2_ emissions

To identify CO₂ emissions (the unit used in this study: Mt) in the digital economy sectors, a comprehensive supply chain perspective is required, encompassing income-, production-, betweenness-, and consumption-based perspectives.

#### 2.1.1 Production-based perspective CO_2_ emissions.

According to IPCC [32], direct CO_2_ emissions from production-based perspective is composed of energy generated by combustion and industrial processes, represented by Eq. (1) and Eq. (2) respectively:


$$E_{g}=\sum_{g=1}^{26}\varepsilon_{g}^{i}c_{g}^{i}$$ (1)

$$E_{k}=\sum_{k=1}^{10}\gamma_{k}p_{k}^{i}$$ (2)

Where $E_{g}$ represents the CO_2_ emissions from combustion of fossil fuels; $\varepsilon_{g}^{i}$ is the emission factor for the g-th type of fuel source consumed by sector i; $c_{g}^{i}$ is the total amount of the g-th type of fuel source consumed by sector i. This study considered 26 fuel sources. $E_{k}$ denotes the CO_2_ emissions from chemical reactions during the production process; $\gamma_{k}$ is the emission factor of the industrial process for producing product k; and $p_{k}^{i}$ is the total quantity of product k produced by sector i. This study selected 10 industrial processes. Therefore, the direct CO_2_ emissions can be represented by Eq. (3):


$$E=E_{g}+E_{k}$$ (3)

#### 2.1.2 Consumption- and income-based perspective CO_2_ emissions.

The environmental input-output model is an extension of the traditional input-output model [33] into the energy and environmental fields. Moreover, the environmental input-output model can link the consumption of final products to energy use across different sectors, thereby facilitating the assessment of environmental pressures within economic systems [34]. It is capable of estimating CO_2_ emissions based on consumption [22] and income [35] through Eq. (4) and Eq. (5) respectively:


$$e={f(I-A)}^{-1}y$$ (4)

$$e^{\prime }={v(I-B)}^{-1}f$$ (5)

Where, e represents embodied CO_2_ emissions from consumption-based perspective; $e^{\prime }$ represents enabled CO_2_ emissions from income-based perspective; y stands for final demand; v denotes initial inputs; I is the identity matrix; and f is direct CO_2_ emissions intensity. Matrices A and B denote the direct domestic input and output coefficient matrix, respectively. Matrix ${\left(I-A\right)}^{-1}$is known as the Leontief inverse, while matrix ${\left(I-B\right)}^{-1}$ is referred to as the Ghosh inverse.

Additionally, Eq. (4) focuses on the embodied CO_2_ emissions driven by the final demand (y) of the sector, while Eq. (5) concentrates on the enabled CO_2_ emissions propelled by the initial inputs (v). Due to the inherent categorization of final demand and initial inputs within the IOT, this study substitutes these classifications and their corresponding values from the table into y in Eq. (4) and v in Eq. (5), to analyze the CO_2_ emission driving source structures of final demand and initial inputs.

#### 2.1.3 Betweenness-based perspective CO_2_ emissions.

Estimation based on betweenness originates from network analysis techniques and can analyze the transmission effect of CO_2_ emissions for a sector. According to existing research [27], the method for estimating betweenness-based CO_2_ emissions for sector i is given by Eq. (6):


$$b_{i}=\;\sum_{s=1}^{n}\sum_{t=1}^{n}\sum_{r=1}^{\infty }\left(q_{r}W\left(s,\;t|k_{1}k_{2},\;\ldots \;,k_{r}\right)\right)$$ (6)

s serves as the starting sector, passing through r sectors (namely $k_{1}$,$k_{2}$…$k_{r}$) to reach the terminal sector t. W denotes the magnitude of environmental pressure generated when sector t pulls on sector s, and $q_{r}$indicates the number of times sector i appears in the supply chain paths.

### 2.2. SPA

SPA [36] decomposes the total emissions of an economy into an infinite number of pathways according to the production system, and identifies critical supply chain paths by their emissions. Specifically, this involves expanding the Leontief inverse matrix in Eq. (4) and the Ghosh inverse matrix in Eq. (5) via Taylor series expansion, leading to CO_2_ footprint from upstream and downstream supply chain paths, as shown in Eq. (7) and Eq. (8):


$$e=f(I+A+A^{2}+A^{3}+...)y=fy+fAy+fA^{2}y+fA^{3}y+...$$ (7)

$$\;e^{\prime }=v(I+B+B^{2}+B^{3}+...)f=vf+vBf+vB^{2}f+vB^{3}f+..$$ (8)

$fA^{n}y$ and $vB^{n}f$ represent the corresponding emissions in all supply chain paths at the *n*th production layer, where the path length is *n*. The number of supply chain paths increases dramatically with the rise in the number of production layers [37]. However, studies have indicated that the cumulative energy consumption significantly increases in the first four layers [38], but the contribution of higher-order pathways is minimal [39]. Therefore, this study selects the first four layers of upstream and downstream supply chains for analysis, with emissions accounting for 92.8% and 97.6% of the total emissions, respectively.

### 2.3. Data sources and processing

The CO_2_ satellite accounts came from the China Emissions Accounts and Datasets [40,41]. And the latest 2017 Input-Output Table (IOT) of Zhejiang Province (for more details, please see Table S1) used in this study was sourced from the Zhejiang Provincial Bureau of Statistics.

In terms of data processing, this study took into account the emission reduction effects of the digital economy sectors. Therefore, following methods outlined in relevant research [42], the emission reduction effects were excluded, restoring the direct CO_2_ emissions of each sector in the CO_2_ satellite accounts. Subsequently, according to [25] and [43], the digital economy content in the IOT and the CO_2_ satellite accounts was separated out into CIDE and the industrial digitalization sector (IDS).

In the IOT, the separation of digital economy content involves column and row splitting, with both approaches following the same principle and requiring the application of Eq. (9) and Eq. (10) to isolate the content of CIDE and IDS, respectively.


$$D_{CIDE}=D_{t}+\sum {(D}_{p}p_{GDP})$$ (9)

$$D_{IDS}=\sum {(D}_{np}p_{r})$$ (10)

Taking the column splitting of intermediate flows as an example, $D_{CIDE}$ represents the intermediate flow of CIDE for each column. $D_{t}$ and $D_{p}$ are the flows on the input side that are entirely or partially associated with CIDE for each column, respectively, with detailed sector classification available in the notes of Table 1. $p_{GDP}$ is the proportion of added value of CIDE to GDP in the given year. $D_{IDS}$ represents the flow of IDS for each column. $D_{np}$ is the flow on the input side for departments excluding those entirely associated with CIDE. $p_{r}$ is the digital economy penetration rate of the department on the input side of each column, which is derived from the digital economy penetration rates reported in [44]. After removing the above two parts of digital economy content from the flows in each column, and separately listing them in rows, the operation of column splitting is completed. Row splitting follows a similar process.

**Table 1:** Overview of the 42 sectors in Zhejiang.

Serial No.	Sector Name	Serial No.	Sector Name
1	Agricultural, forestry, livestock, fishery products and services	22	Metal products, machinery, and equipment repair services
2	Coal mining and selection products	23	Production and supply of electricity, heat
3	Petroleum and natural gas extraction products	24	Gas production and supply
4	Metal mine selection products	25	Water production and supply
5	Non-metallic mineral and other mine selection products	26	Construction*
6	Food and tobacco	27	Wholesale and retail*
7	Textiles	28	Transportation, storage, and postal services
8	Textile, footwear, leather, and feather products	29	Accommodation and catering
9	Wood processing products and furniture	30	Finance*
10	Paper, printing, and educational, cultural, sports goods*	31	Real estate
11	Petroleum, coke products, and nuclear fuel processing	32	Leasing and business services*
12	Chemical products*	33	Research and experimental development*
13	Non-metallic mineral products	34	Comprehensive technical services*
14	Metal smelting and rolling products	35	Water conservancy, environment, and public facility management
15	Metal products	36	Resident services, repair, and other services*
16	General equipment*	37	Education
17	Specialized equipment*	38	Health and social work
18	Transportation equipment	39	Culture, sports, and entertainment*
19	Electrical machinery and equipment*	40	Public administration, social security, and social organizations
20	Instruments and meters*	41	Core industries of digital economy
21	Other manufacturing products and waste	42	Industrial digitalization

Notes: (1) An asterisk (*) indicates that part of this sector in the original IOT belongs to the core industries of the digital economy; (2) In the original IOT, the sectors “communications equipment, computers, and other electronic devices” and “information transmission, software, and information technology services” have been fully classified under the core industries of the digital economy.

As the scope of this study is restricted to production activities in Zhejiang Province, China, we followed previous research [45] to segregate import inflow data from the table. After all processing, the gross value added for CIDE and IDS identified in this study differed by 1.52% and 4.42%, respectively, from pertinent publicly available data [46,47]. Moreover, the process for separating the digital economy content from the CO_2_ satellite account was guided by Eq. (7) and Eq. (8). Ultimately, this allowed us to compile a IOT and a CO_2_ emissions inventory for new 42 sectors in Zhejiang Province (for more details, please see Tables S2 and S3), with the sector numbers and names detailed in Table 1.

## 3. Results

### 3.1. CO_2_ emissions of digital economy sectors: A supply chain perspective

#### 3.1.1 Comparison of CO_2_ emission rankings based on four perspectives.

**Fig 3:**
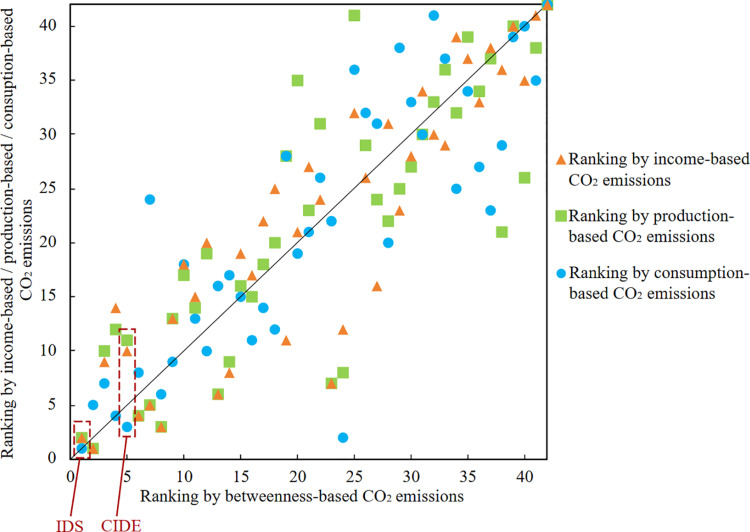
CO_2_ emission rankings from multiple perspectives.

Fig 3 shows the rankings of 42 sectors in terms of CO₂ emissions from the perspectives of income, production, consumption, and betweenness (for more details, please see Table S4). It is evident that the digital economy sectors rank high among the 42 sectors under all four analytical frameworks. This indicates the urgency of reducing CO_2_ emissions through supply chain management, which should be given priority by policymakers. The results highlight that, from an income perspective, IDS ranks second, while CIDE ranks tenth. From a production perspective, IDS ranks second, and CIDE ranks eleventh. From a betweenness perspective, IDS ranks first, and CIDE ranks fifth. From a consumption perspective, the CO_2_ emissions of IDS and CIDE rank first and third, respectively. These findings suggest that the digital economy sectors drive more CO_2_ emissions as both an intermediate and a final consumption sector within the supply chains. Moreover, compared to CIDE, IDS contributes to higher levels of CO_2_ emissions.

#### 3.1.2 The structure of CO_2_ emissions drivers.

The IOT categorizes and quantifies final demand and initial inputs, aiding our analysis of the driving sources’ structure for embodied and enabled CO_2_ emissions in the digital economy sectors’ supply chains. The research findings will enhance the effectiveness of resource allocation for emission reduction policies, allowing for precise optimization of consumption and investment behaviors of the supply chains, thus achieving efficient CO_2_ mitigation.

**Fig 4:**
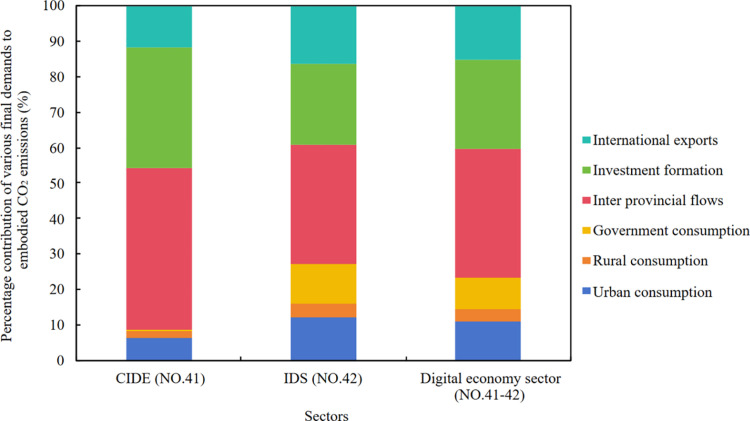
Percentage contribution of various final demands to the embodied CO_2_ emissions.

Firstly, the calculation method from a consumption perspective quantifies the embodied CO_2_ emissions pulled by final demand. Final demand can be categorized according to the components specified in the IOT: urban consumption, rural consumption, government consumption, inter provincial flows, investment formation, and international exports. Fig 4 displays the percentage breakdown of embodied CO_2_ emissions driven by these final demands of the digital economy sectors’ supply chains.

At the macro level, inter-provincial flows stand out as the leading contributor to embodied CO_2_ emissions, followed by investment formation and international exports. Upon closer inspection of specific sectors, the structure of driving sources for CIDE and IDS exhibits broad similarities. However, it is noteworthy that inter provincial flows and investment formation contribute more significantly to the CO_2_ emissions of CIDE, accounting for 45.63% and 33.94% respectively, compared to 33.78% and 22.81% for IDS. This observation suggests that targeted policy designs for reducing emissions of the digital economy sectors should focus on key drivers such as inter provincial flows, investment formation, and international exports.

**Fig 5:**
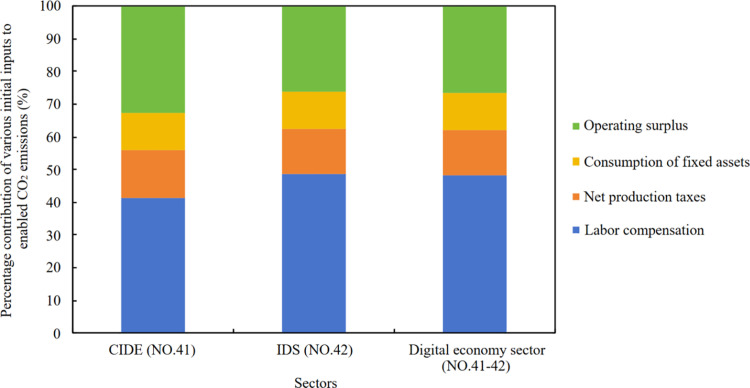
Percentage contribution of various initial inputs to the income-based CO_2_ emissions.

Secondly, the calculation method based on an income perspective can quantify the enabled CO₂ emissions driven by initial inputs. Since this study focuses on departments within the province, data related to imports and inflows from other provinces domestically have been removed during data processing. These initial inputs can be classified according to the components specified in the IOT: labor compensation, net production taxes, consumption of fixed assets, and operating surplus. Fig 5 shows the proportions of enabled CO₂ emissions in the digital economy sectors’ supply chains that are attributable to different initial inputs.

There is consistency in the contribution structure of initial inputs to enabling CO₂ emissions across the digital economy sectors, with labor compensation being the primary contributor, followed by operating surplus, net production taxes, and consumption of fixed assets. They drive the total CO_2_ emissions contribution ratios of the two digital economy sectors at 48.17%, 26.70%, 14.06%, and 11.07%. Labor compensation, as the primary driving source, has a significantly higher contribution ratio than other source, highlighting its importance for CO_2_ reduction.

### 3.2. Analysis of the pulling relationships within upstream supply chain paths

**Fig 6:**
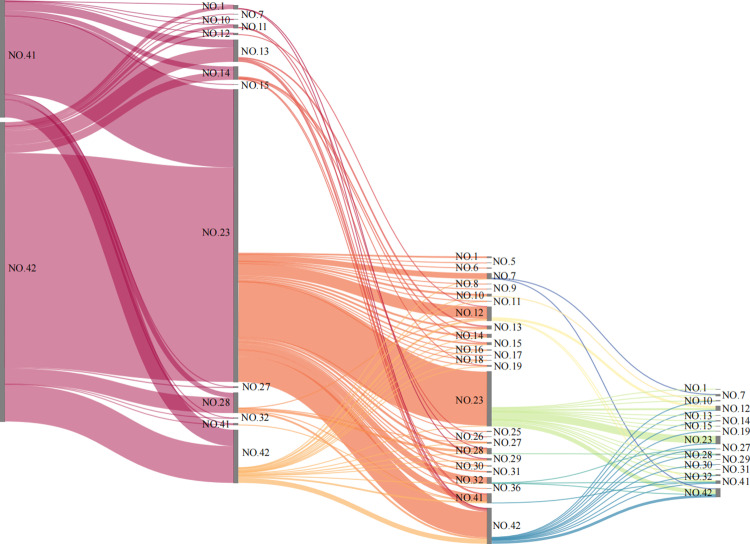
The top 100 CO_2_ emission upstream supply chain paths of digital economy sectors (unit: Mt).

The digital economy sectors exert a pulling effect on CO_2_ emissions within their upstream supply chain paths. SPA can identify the critical CO₂ emission paths with more pronounced pulling effects, and the outcomes will aid in formulating targeted green consumption guidance policies for consumption of key digital economy products. Fig 6 illustrates the top 100 CO_2_ emission upstream supply chain paths of the digital economy sectors (for more details, please see Table S5), primarily characterized by short paths concentrated at lengths of 1 and 2 steps.

Table 2 presents the top 5 CO_2_ emission upstream supply chain paths of the digital economy sectors. For CIDE (NO.41), the supply chain path from “electric power and heat production and supply (NO. 23)” transmits the highest amount of CO_2_, totaling 6.8 Mt, which represents 24.64% of this sector’s total emissions. Similarly, in IDS (NO. 42), the same supply chain path leads in CO_2_ transmission, with 21.91 Mt, accounting for 20.67% of its total emissions. Among these, the upstream chains with the highest CO_2_ emissions are uniformly those that flow directly from electric power and heat production and supply (NO.23) to digital economy sectors (NO.41–42), characterized by short paths and high coverage of CO_2_ emissions. Second to this are the upstream chain paths that flow directly from IDS (NO.42) to digital economy sectors (NO.41–42).

**Table 2:** The top 5 CO_2_ emission upstream supply chain paths of digital economy sectors.

Sectors	Paths	Emissions (Mt)	Coverage (%)
CIDE (NO.41)	NO.23 → NO.41	6.80	24.64
	NO.42 → NO.41	1.91	6.92
	NO.23 → NO.23 → NO.41	1.45	5.26
	NO.13 → NO.41	1.10	3.99
	NO.42 → NO.23 → NO.41	0.99	3.59
IDS (NO.42)	NO.23 → NO.42	21.91	20.67
	NO.42 → NO.42	4.73	4.46
	NO.23 → NO.23 → NO.42	4.68	4.41
	NO.42 → NO.23 → NO.42	2.45	2.31
	NO.28 → NO.42	2.05	1.93

Notably, two upstream supply chain paths have entered the high-carbon sequences of their respective sectors, namely “non-metallic mineral products (NO.13) ◊ CIDE (NO.41)” and “transportation, warehousing, and postal services (NO.28) ◊ industrial digitalization sector (NO.42).” Relevant management departments should pay close attention to these and fully consider their decarbonization tasks.

### 3.3. Analysis of the promoting relationships within downstream supply chain paths

**Fig 7:**
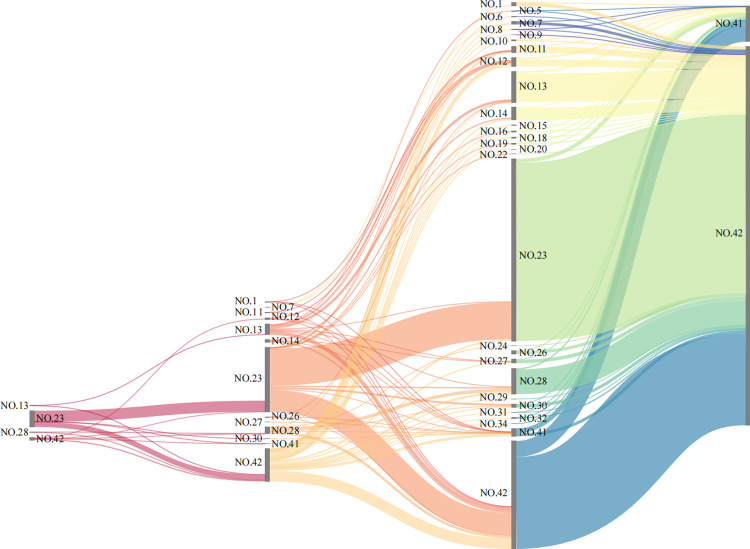
The top 100 CO_2_ emission downstream supply chain paths of the digital economy sectors (unit: Mt).

The digital economy sectors exert a promoting effect on CO_2_ emissions within their downstream supply chain paths. Identifying the key CO₂ emission paths with more pronounced driving effects will help in adjusting the initial input sectors in the critical supply chain paths. Fig 7 illustrates the top 100 CO_2_ emission downstream chain paths of the digital economy sectors (for more details, please see Table S6), where high-carbon paths predominantly consist of short chains, concentrated at lengths of 1 or 2 steps.

Table 3 showcases the top 5 CO_2_ emission downstream chain paths. For CIDE (NO.41), the downstream path to IDS (NO.42) transmits the most enabled CO_2_, totaling 0.90 Mt and accounting for 14.82% of this sector’s total emissions. Similarly, for IDS (NO.42), the downstream path to electric power and heat production and supply (NO. 23) transmits 12.98 Mt of enabled CO_2_, representing 14.87% of its total emissions. Among these, the digital economy sectors primarily promote CO_2_ emissions in downstream chain paths between themselves and the energy sector (NO.23), as well as internally (NO.41–42), highlighting the importance of optimizing capital investment structures in the energy and digital economy sectors for reducing emissions along the downstream chain paths. Secondarily, they promote CO_2_ emissions in downstream chain paths with non-metallic mineral products (NO.13).

**Table 3:** The top 5 CO_2_ emission downstream supply chain paths of the digital economy sectors.

Sectors	Paths	Emissions (Mt)	Coverage (%)
CIDE (NO.41)	NO.41 → NO.42	0.90	14.82
	NO.41 → NO.23	0.29	4.81
	NO.41 → NO.42 → NO.23	0.25	4.15
	NO.41 → NO.13	0.19	3.11
	NO.41 → NO.41	0.18	2.91
IDS (NO.42)	NO.42 → NO.23	12.98	14.87
	NO.42 → NO.42	5.18	5.93
	NO.42 → NO.23 → NO.23	2.77	3.17
	NO.42 → NO.13	2.44	2.79
	NO.42 → NO.28	1.61	1.84

At the individual sector level, CIDE (NO.41) tends to generate its promotional effect on CO_2_ emissions in downstream chain paths mostly through direct consumption by IDS (NO.42), whereas it does so mainly through direct consumption by the energy sector (NO.23).

## 4. Discussion

### 4.1. High-CO_2_ emission characteristics of digital economy sectors across the supply chain

The digital economy sectors rank relatively high in CO_2_ emissions from the perspectives of consumption, betweenness, income, and production among all sectors, highlighting the importance of reducing emissions in the digital economy sectors and their supply chains.

Particularly, the digital economy sectors lead in emission rankings when assessed from a consumption perspective, indicating that, overall, the digital economy sectors pull a considerable amount of CO_2_ emissions within their supply chains through final demand, aligning with findings by [13]. The primary reasons for this are twofold: firstly, digital economy products are generally linked to upstream energy sectors, and given that coal combustion constitutes 52.4% of Zhejiang Province’s energy mix [48], the limited cleanliness of energy sources results in higher CO_2_ emissions being drawn through electricity usage. Secondly, CIDE (NO.41) and IDS (NO.42) depend heavily on products such as non-metallic mineral products and upstream foundational digital economy goods, thereby contributing significantly to CO_2_ emissions through consumption.

For CO_2_ emissions related to IDS (NO.42), in addition to the final demand that has a generally significant pull effect, there is also a substantial impact from betweenness, consistent with conclusions by [27]. This is because the sector is exceptionally intertwined with its upstream and downstream sectors; its products and services consume various goods from the digital economy sectors and energy sectors upstream, while downstream they are widely applied across domains such as green technology innovation [49], clean energy development [50], and consumption structure upgrades [51] leading to the generation of substantial CO_2_ emissions based on betweenness within the supply chains.

Moreover, for the driving sources, under the consumption-based measurement perspective, inter-provincial flows emerge as the leading source driving embodied CO_2_ emissions within the supply chains of Zhejiang’s digital economy sectors, primarily associated with the province’s overall CO_2_ emission transfers and CO_2_ spillover in the digital industry. Firstly, due to thriving inter-provincial trade, Zhejiang became the third-largest province for inter-provincial CO_2_ emission transfers in 2017 [52], making inter-provincial flows a crucial type of final demand for the province. Secondly, at the sectoral level, research indicates that digital industries in coastal provinces, including Zhejiang, exhibit significant carbon spillovers [53], reflecting the current situation where Zhejiang’s digital economy sectors, due to their extensive involvement in the supply chains of out-of-province sectors such as power, chemicals, and non-metallic and metallic products, face substantial demands for domestic outflows, thus drawing large amounts of embodied CO_2_ emissions.

From the income-based measurement perspective, labor compensation is the primary driver of enabled CO_2_ emissions in the supply chains of the digital economy sectors, which aligns with the findings of Zhou et al. [26] regarding the national digital economy in 2017. This is because the development of the digital economy sectors has increased employment, particularly in research and development and innovation positions. The research by Qiu et al. [54] on the digital empowerment of manufacturing enterprises provides strong support for this observation. However, behind the growth in employment is an increase in labor compensation investment in the initial input sector. Therefore, with the significant growth of employment in the digital economy sectors in 2018 [55], labor compensation investment has increased substantially, expanding the scale of downstream. Consequently, it is unsurprising that labor compensation inputs are associated with a significant amount of downstream enabled CO_2_ emissions.

### 4.2. Upstream and downstream CO_2_ emission characteristics of digital economy sectors

The characteristics of CO_2_ emissions related with the digital economy sectors vary across different types of supply chains and specific sectors. The analysis below focuses on the share of CO_2_ emissions from the top 5 high-carbon supply chain paths presented in Table 4. Primarily, from an overarching perspective, compared to downstream supply chain paths, the CO_2_ emissions from upstream supply chain paths stimulated by the digital economy sectors are more concentrated. This is due to their primary consumption of products from the energy sector and their own sector upwards, and their distribution across various industries downwards.

**Table 4:** CO_2_ emissions in the top 5 high-carbon supply chain paths of the digital economy sectors.

Sectors	Upstream supply chain paths		downstream supply chain paths	
Emissions (Mt)	Coverage (%)	Emissions (Mt)	Coverage (%)
CIDE (NO.41)	12.24	44.39	1.81	29.79
IDS (NO.42)	35.81	33.79	24.97	28.60

Secondly, in detail, CO_2_ emissions from upstream and downstream supply chain paths driven by CIDE (NO.41) are more concentrated, whereas those driven by IDS (NO.42) are relatively dispersed. The reason lies in the fact that CIDE (NO.41), representing the development of digital product manufacturing, digital product trading, and digital information technology, consumes mainly manufacturing materials, finished digital products, and energy products upwards, and flows mainly towards IDS and energy production sectors downwards, hence the relative concentration. Meanwhile, IDS (NO.42), having permeated various aspects of social production in the form of information technology, has significantly enhanced the value chains of various industries [56], and thus triggers broader up-and-downstream CO_2_ emissions through input-output relationships.

Regarding the upstream supply chain paths of the digital economy sectors, the high-carbon paths are predominantly short step-length paths. This is partly due to the general dependence and direct consumption of products from the electric power and heat production and supply (NO.23) by the digital economy sectors, meaning the most CO_2_ emissions are pulled during direct electricity usage. Additionally, the products and services of the digital economy sectors rely on products from sectors such as non-metallic mineral products (NO.13), metal smelting and rolling processing products (NO.14), transportation, warehousing, and postal services (NO.28), leading to increased emissions during the direct consumption process from these sectors’ production activities.

Concerning the downstream supply chain paths of the digital economy sectors, the high-carbon paths continue to be predominantly short in length. However, uniquely, due to the requirement to embed a substantial amount of products from CIDE (NO.41) into existing industrial systems during the process of industrial digitalization, the downstream chain path with the highest CO_2_ emission coverage rate from CIDE (NO.41) is the direct flow towards IDS (NO.42); while that from IDS (NO.42) is the direct flow towards electric power and heat production and supply (NO.23), a view similar to that advocated by [13]. Consequently, a considerable amount of CO_2_ emissions stimulated by the internal product flows within the supply chains of the digital economy sectors is noteworthy, demanding attention from current emission reduction policies.

## 5. Conclusions and policy implications

This research analyzed the CO_2_ emissions related to digital economy sectors from whole supply chain perspectives, using Zhejiang as a case. It identified the structure of final demands that pull embodied CO_2_ emissions and initial inputs that promote enabled CO_2_ emissions. It also determined the critical driving relationships for CO_2_ emissions related to the digital economy sectors from both upstream and downstream supply chain paths. The following are the major conclusions and policy implications derived:

(1) The two digital economy sectors exhibit slight differences in their CO_2_ emissions rankings across four perspectives of their supply chains. CIDE ranks highest from consumption-based perspective, while IDS ranks highest from both consumption-based and betweenness-based perspectives. This indicates that the digital economy sectors drive the majority of CO_2_ emissions in its supply chains through final demand. Consequently, policies should prioritize the optimization of consumption structures, particularly the energy consumption structure. Although China’s energy consumption structure has improved over the past 40 years [57], further efforts are still needed. For instance, the establishment of green procurement and usage lists could be implemented, accompanied by fiscal subsidies or tax reductions for enterprises that consistently select products with low embodied CO₂ emissions and clean energy sources. Furthermore, the unique scenario in which IDS also ranks prominently in the betweenness perspective warrants policy attention. A rigorous evaluation of CO₂ emissions associated with digital economy products based on intermediary activities should be conducted, and additional taxes should be levied on the production of products with excessively high emissions.
(2) Inter-provincial flows are the main source driving the embodied CO₂ emissions in the digital economy sectors’ supply chains, while labor compensation is the primary driving source of its enabled CO₂ emissions. Therefore, policies should enhance interprovincial cooperation in CO₂ emission reduction. From a national perspective, the establishment of a unified digital economy carbon market is recommended to facilitate cross-regional consumption-based CO₂ accounting and its corresponding scientific taxation system. This would incentivize digital economy enterprises to expand green consumption practices. Regarding labor compensation, policies should aim to increase the proportion of supply chains with low enabled emissions to achieve efficient relative emission reductions. Feasible measures include implementing fiscal subsidies and talent recruitment programs for enterprises with low enabled emissions, thereby assisting them in increasing employment opportunities and expanding labor compensation investments.
(3) High-carbon upstream and downstream supply chain paths driven by the digital economy sectors predominantly feature short path lengths, with power and heat production and supply, as well as industrial digitalization, playing crucial roles. Therefore, it is recommended to establish a carbon trading market between the upstream chains of the digital economy sectors and key primary production sectors. Digital economy-specific carbon emission quotas should be allocated to each sector involved, and trading rules should be refined to encourage enterprises to focus on and reduce their carbon emissions associated with digital economy. For emissions driven by internal product flows within downstream chains, the government should refine its carbon tax system based on income-based responsibility. Specifically, enterprises producing products with excessively high income-based CO₂ emissions should be subject to proportional carbon taxes. This would compel relevant companies to consider downstream CO₂ emissions in their collaborations and opt for cleaner buyers.

## Nomenclature

CEI CO_2_ emissions intensity

CIDE the core industry sector of the digital economy

IDS the industrial digitalization sector

IOT Input-output table

E Direct CO_2_ emissions

$E_{g}$ the CO_2_ emissions from combustion of fossil fuels

$E_{k}$ the CO_2_ emissions from chemical reactions during the production process

$\varepsilon_{g}^{i}$ the emission factor for the g-th type of fuel source consumed by sector i

$c_{g}^{i}$ the total amount of the g-th type of fuel source consumed by sector i

$\gamma_{k}$ the emission factor of the industrial process for producing product k

$p_{k}^{i}$ the total quantity of product k produced by sector i

e Embodied CO_2_ emissions

$e^{\prime }$ Enabled CO_2_ emissions

y The vector of final demand

v The vector of initial inputs

I Identity matrix

f Direct CEI

A Direct domestic input coefficient matrix

B Direct domestic output coefficient matrix

$b_{i}$ Betweenness-based CO_2_ emissions of sector i

s The starting sector

$k_{r}$ The middle sector

t The terminal sector

$q_{r}$ The number of times sector i appears in the supply chain path

n The length of supply chain path

$D_{CIDE}$ The matrix of the core industry sector of the digital economy

$D_{t}$ The matrix entirely belongs to the core industry sector of the digital economy

$D_{p}$ The matrix partially belongs to the core industry sector of the digital economy

$p_{GDP}$ The share of the core industry sector of the digital economy in GDP

$D_{IDS}$ The matrix of the industrial digitalization sector

$D_{np}$ The IOT matrices after removing sectors entirely belonging to the core industries of the digital economy

$p_{r}$ The penetration of digital economy sectors

## Supporting information

S1 Table 2017 Zhejiang Province Input-Output Table (Original Table). (XLSX)

S2 Table 2017 Zhejiang Province Input-Output Table (Adjusted Table). (XLSX)

S3 Table Direct CO_2_ Emission and Coefficient of 42 Sectors in Zhejiang Province. (DOCX)

S4 Table CO_2_ Emission ranks of 42 Sectors in Zhejiang Province. (DOCX)

S5 Table The top 100 CO_2_ emission upstream supply chains in the digital economy sectors. (DOCX)

S6 Table The top 100 CO_2_ emission downstream supply chains in the digital economy sectors. (DOCX)
