# Supplementary material for: Mitigating CO2 emissions associated with digital economy sectors through whole supply chain management
Source: PLoS One. 2025 May 20;20(5):e0323350. doi: 10.1371/journal.pone.0323350 (PMC12091893; doi:10.1371/journal.pone.0323350)
Supplement: S3 Table — (DOCX) [file pone.0323350.s003.docx]

# Supplementary data for:

# Mitigating CO_2_ emissions associated with digital economy sectors through whole supply chain management

**Table S3. Direct CO_2_ Emission and Coefficient of 42 Sectors in Zhejiang Province.**

| Serial NO. | Sectors | Coefficient  (Mt /10000 yuan) | Direct CO_2_ Emission  (Mt) |
| --- | --- | --- | --- |
| 1 | Agricultural, forestry, livestock, fishery products and services | 2.2647E-07 | 7.202791878 |
| 2 | Coal mining and selection products | 3.29278E-06 | 0.122284264 |
| 3 | Petroleum and natural gas extraction products | 0 | 0 |
| 4 | Metal mine selection products | 4.47204E-08 | 0.008152284 |
| 5 | Non-metallic mineral and other mine selection products | 6.76692E-08 | 0.130436548 |
| 6 | Food and tobacco | 1.85957E-08 | 0.489137056 |
| 7 | Textiles | 3.97915E-08 | 2.298944162 |
| 8 | Textile, footwear, leather, and feather products | 9.00704E-09 | 0.391309645 |
| 9 | Wood processing products and furniture | 8.27053E-09 | 0.154893401 |
| 10 | Paper, printing, and educational, cultural, sports goods | 2.61242E-08 | 0.855350254 |
| 11 | Petroleum, coke products, and nuclear fuel processing | 2.80528E-07 | 5.347898477 |
| 12 | Chemical products | 3.00459E-08 | 2.947005076 |
| 13 | Non-metallic mineral products | 1.47282E-06 | 31.76129949 |
| 14 | Metal smelting and rolling products | 3.91444E-07 | 12.05722843 |
| 15 | Metal products | 1.84036E-08 | 0.570659898 |
| 16 | General equipment | 1.84303E-08 | 0.848162437 |
| 17 | Specialized equipment | 8.62874E-09 | 0.165319797 |
| 18 | Transportation equipment | 1.6618E-08 | 0.701096447 |
| 19 | Electrical machinery and equipment | 8.111E-09 | 0.438456853 |
| 20 | Instruments and meters | 6.4962E-09 | 0.057502538 |
| 21 | Other manufacturing products and waste | 9.11294E-09 | 0.138588832 |
| 22 | Metal products, machinery, and equipment repair services | 6.2532E-08 | 0.048913706 |
| 23 | Production and supply of electricity, heat | 4.7078E-06 | 193.9183858 |
| 24 | Gas production and supply | 8.19008E-09 | 0.024456853 |
| 25 | Water production and supply | 4.38566E-09 | 0.008152284 |
| 26 | Construction | 4.28867E-08 | 4.147370558 |
| 27 | Wholesale and retail | 5.27721E-08 | 2.981147208 |
| 28 | Transportation, storage, and postal services | 4.30036E-07 | 19.16767513 |
| 29 | Accommodation and catering | 1.67749E-09 | 0.03813198 |
| 30 | Finance | 2.89581E-09 | 0.080862944 |
| 31 | Real estate | 4.8723E-09 | 0.05719797 |
| 32 | Leasing and business services | 2.93881E-08 | 1.040436548 |
| 33 | Research and experimental development | 7.21895E-09 | 0.016172589 |
| 34 | Comprehensive technical services | 4.74458E-09 | 0.059299492 |
| 35 | Water conservancy, environment, and public facility management | 1.23199E-08 | 0.05084264 |
| 36 | Resident services, repair, and other services | 1.75933E-08 | 0.107817259 |
| 37 | Education | 3.03606E-08 | 0.209725888 |
| 38 | Health and social work | 1.06483E-09 | 0.01906599 |
| 39 | Culture, sports, and entertainment | 2.13275E-09 | 0.010781726 |
| 40 | Public administration, social security, and social organizations | 2.37114E-09 | 0.04448731 |
| 41 | Core industries of digital economy | 1.94721E-08 | 2.771229695 |
| 42 | Industrial digitalization | 2.13525E-07 | 78.36508503 |
