# Supplementary material for: Mitigating CO2 emissions associated with digital economy sectors through whole supply chain management
Source: PLoS One. 2025 May 20;20(5):e0323350. doi: 10.1371/journal.pone.0323350 (PMC12091893; doi:10.1371/journal.pone.0323350)
Supplement: S4 Table — (DOCX) [file pone.0323350.s004.docx]

# Supplementary data for:

# Mitigating CO_2_ emissions associated with digital economy sectors through whole supply chain management

**Table S4. CO_2_ Emission ranks of 42 Sectors in Zhejiang Province.**

| Serial NO. | Sectors | Production-based rank | Consumption-based rank | Income-based rank | Betweenness-based rank |
| --- | --- | --- | --- | --- | --- |
| 1 | Agricultural, forestry, livestock, fishery products and services | 6 | 16 | 6 | 13 |
| 2 | Coal mining and selection products | 26 | 40 | 35 | 40 |
| 3 | Petroleum and natural gas extraction products | 42 | 41 | 42 | 42 |
| 4 | Metal mine selection products | 40 | 39 | 40 | 39 |
| 5 | Non-metallic mineral and other mine selection products | 25 | 38 | 23 | 29 |
| 6 | Food and tobacco | 18 | 14 | 22 | 17 |
| 7 | Textiles | 12 | 4 | 14 | 4 |
| 8 | Textile, footwear, leather, and feather products | 20 | 12 | 25 | 18 |
| 9 | Wood processing products and furniture | 23 | 21 | 27 | 21 |
| 10 | Paper, printing, and educational, cultural, sports goods | 14 | 13 | 15 | 11 |
| 11 | Petroleum, coke products, and nuclear fuel processing | 7 | 22 | 7 | 23 |
| 12 | Chemical products | 10 | 7 | 9 | 3 |
| 13 | Non-metallic mineral products | 3 | 6 | 3 | 8 |
| 14 | Metal smelting and rolling products | 5 | 24 | 5 | 7 |
| 15 | Metal products | 17 | 18 | 18 | 10 |
| 16 | General equipment | 15 | 11 | 17 | 16 |
| 17 | Specialized equipment | 22 | 20 | 31 | 28 |
| 18 | Transportation equipment | 16 | 15 | 19 | 15 |
| 19 | Electrical machinery and equipment | 19 | 10 | 20 | 12 |
| 20 | Instruments and meters | 30 | 30 | 34 | 31 |
| 21 | Other manufacturing products and waste | 24 | 31 | 16 | 27 |
| 22 | Metal products, machinery, and equipment repair services | 33 | 42 | 30 | 32 |
| 23 | Production and supply of electricity, heat | 1 | 5 | 1 | 2 |
| 24 | Gas production and supply | 36 | 37 | 29 | 33 |
| 25 | Water production and supply | 41 | 36 | 32 | 25 |
| 26 | Construction | 8 | 2 | 12 | 24 |
| 27 | Wholesale and retail | 9 | 17 | 8 | 14 |
| 28 | Transportation, storage, and postal services | 4 | 8 | 4 | 6 |
| 29 | Accommodation and catering | 35 | 19 | 21 | 20 |
| 30 | Finance | 28 | 28 | 11 | 19 |
| 31 | Real estate | 31 | 26 | 24 | 22 |
| 32 | Leasing and business services | 13 | 9 | 13 | 9 |
| 33 | Research and experimental development | 38 | 35 | 41 | 41 |
| 34 | Comprehensive technical services | 29 | 32 | 26 | 26 |
| 35 | Water conservancy, environment, and public facility management | 32 | 25 | 39 | 34 |
| 36 | Resident services, repair, and other services | 27 | 33 | 28 | 30 |
| 37 | Education | 21 | 29 | 36 | 38 |
| 38 | Health and social work | 37 | 23 | 38 | 37 |
| 39 | Culture, sports, and entertainment | 39 | 34 | 37 | 35 |
| 40 | Public administration, social security, and social organizations | 34 | 27 | 33 | 36 |
| 41 | Core industries of digital economy | 11 | 3 | 10 | 5 |
| 42 | Industrial digitalization | 2 | 1 | 2 | 1 |
