# Supplementary material for: Mitigating CO2 emissions associated with digital economy sectors through whole supply chain management
Source: PLoS One. 2025 May 20;20(5):e0323350. doi: 10.1371/journal.pone.0323350 (PMC12091893; doi:10.1371/journal.pone.0323350)
Supplement: S5 Table — (DOCX) [file pone.0323350.s005.docx]

# Supplementary data for:

# Mitigating CO_2_ emissions associated with digital economy sectors through whole supply chain management

**Table S5. The top 100 CO_2_ emission upstream supply chains in the digital economy sectors.**

| Emissions (Mt) | Paths | Emissions (Mt) | Paths |
| --- | --- | --- | --- |
| 6.799725 | NO.23 🡪 NO.41 | 21.9088 | NO.23 🡪 NO.42 |
| 1.908336 | NO.42 🡪 NO.41 | 4.726333 | NO.42 🡪 NO.42 |
| 1.450955 | NO.23 🡪 NO.23 🡪 NO.41 | 4.674995 | NO.23 🡪 NO.23 🡪 NO.42 |
| 1.101471 | NO.13 🡪 NO.41 | 2.450073 | NO.42 🡪 NO.23 🡪 NO.42 |
| 0.989258 | NO.42 🡪 NO.23 🡪 NO.41 | 2.050894 | NO.28 🡪 NO.42 |
| 0.749539 | NO.41 🡪 NO.23 🡪 NO.41 | 1.974291 | NO.13 🡪 NO.42 |
| 0.654036 | NO.14 🡪 NO.41 | 1.08624 | NO.14 🡪 NO.42 |
| 0.585929 | NO.28 🡪 NO.41 | 1.057306 | NO.12 🡪 NO.23 🡪 NO.42 |
| 0.463836 | NO.12 🡪 NO.23 🡪 NO.41 | 0.997571 | NO.23 🡪 NO.23 🡪 NO.23 🡪 NO.42 |
| 0.380133 | NO.32 🡪 NO.23 🡪 NO.41 | 0.652044 | NO.7 🡪 NO.23 🡪 NO.42 |
| 0.309611 | NO.23 🡪 NO.23 🡪 NO.23 🡪 NO.41 | 0.562194 | NO.1 🡪 NO.42 |
| 0.21341 | NO.42 🡪 NO.42 🡪 NO.41 | 0.528549 | NO.42 🡪 NO.42 🡪 NO.42 |
| 0.212932 | NO.41 🡪 NO.41 | 0.526044 | NO.32 🡪 NO.23 🡪 NO.42 |
| 0.211092 | NO.42 🡪 NO.23 🡪 NO.23 🡪 NO.41 | 0.522807 | NO.42 🡪 NO.23 🡪 NO.23 🡪 NO.42 |
| 0.210357 | NO.41 🡪 NO.42 🡪 NO.41 | 0.425244 | NO.11 🡪 NO.42 |
| 0.15994 | NO.41 🡪 NO.23 🡪 NO.23 🡪 NO.41 | 0.297242 | NO.28 🡪 NO.28 🡪 NO.42 |
| 0.128093 | NO.10 🡪 NO.23 🡪 NO.41 | 0.273993 | NO.42 🡪 NO.42 🡪 NO.23 🡪 NO.42 |
| 0.121416 | NO.41 🡪 NO.13 🡪 NO.41 | 0.269295 | NO.41 🡪 NO.23 🡪 NO.42 |
| 0.121128 | NO.14 🡪 NO.23 🡪 NO.41 | 0.26217 | NO.28 🡪 NO.23 🡪 NO.42 |
| 0.118226 | NO.13 🡪 NO.23 🡪 NO.41 | 0.246328 | NO.1 🡪 NO.23 🡪 NO.42 |
| 0.110629 | NO.42 🡪 NO.42 🡪 NO.23  NO.41 | 0.237425 | NO.12 🡪 NO.12 🡪 NO.23 🡪 NO.42 |
| 0.110319 | NO.29 🡪 NO.23 🡪 NO.41 | 0.229353 | NO.42 🡪 NO.28 🡪 NO.42 |
| 0.109047 | NO.41 🡪 NO.42 🡪 NO.23 🡪 NO.41 | 0.225613 | NO.12 🡪 NO.23 🡪 NO.23 🡪 NO.42 |
| 0.104157 | NO.12 🡪 NO.12 🡪 NO.23 🡪 NO.41 | 0.220786 | NO.42 🡪 NO.13 🡪 NO.42 |
| 0.102029 | NO.1 🡪 NO.41 | 0.21191 | NO.13 🡪 NO.23 🡪 NO.42 |
| 0.10199 | NO.11 🡪 NO.41 | 0.20927 | NO.12 🡪 NO.42 |
| 0.098975 | NO.12 🡪 NO.23 🡪 NO.23  NO.41 | 0.201173 | NO.14 🡪 NO.23 🡪 NO.42 |
| 0.097498 | NO.7 🡪 NO.23 🡪 NO.41 | 0.199254 | NO.10 🡪 NO.23 🡪 NO.42 |
| 0.097426 | NO.15 🡪 NO.23 🡪 NO.41 | 0.190582 | NO.27 🡪 NO.42 |
| 0.095833 | NO.13 🡪 NO.13 🡪 NO.41 | 0.183952 | NO.12 🡪 NO.42 🡪 NO.42 |
| 0.092605 | NO.42 🡪 NO.28 🡪 NO.41 | 0.174045 | NO.31 🡪 NO.23 🡪 NO.42 |
| 0.091806 | NO.12 🡪 NO.41 | 0.171772 | NO.13 🡪 NO.13 🡪 NO.42 |
| 0.089146 | NO.42 🡪 NO.13 🡪 NO.41 | 0.167626 | NO.29 🡪 NO.23 🡪 NO.42 |
| 0.08492 | NO.28 🡪 NO.28 🡪 NO.41 | 0.164817 | NO.15 🡪 NO.23 🡪 NO.42 |
| 0.082807 | NO.14 🡪 NO.14 🡪 NO.41 | 0.14451 | NO.28 🡪 NO.42 🡪 NO.42 |
| 0.082622 | NO.41 🡪 NO.41 🡪 NO.23 🡪 NO.41 | 0.139136 | NO.7 🡪 NO.23 🡪 NO.23 🡪 NO.42 |
| 0.081114 | NO.32 🡪 NO.23 🡪 NO.23 🡪 NO.41 | 0.137528 | NO.14 🡪 NO.14 🡪 NO.42 |
| 0.080699 | NO.12 🡪 NO.42 🡪 NO.41 | 0.1312 | NO.6 🡪 NO.23 🡪 NO.42 |
| 0.07607 | NO.19 🡪 NO.23 🡪 NO.41 | 0.125903 | NO.7 🡪 NO.7 🡪 NO.23 🡪 NO.42 |
| 0.0749 | NO.28 🡪 NO.23 🡪 NO.41 | 0.121475 | NO.42 🡪 NO.14 🡪 NO.42 |
| 0.072095 | NO.41 🡪 NO.14 🡪 NO.41 | 0.118239 | NO.42 🡪 NO.12 🡪 NO.23 🡪 NO.42 |
| 0.067298 | NO.27 🡪 NO.41 | 0.11251 | NO.23 🡪 NO.42 🡪 NO.42 |
| 0.064587 | NO.41 🡪 NO.28 🡪 NO.41 | 0.112249 | NO.32 🡪 NO.23 🡪 NO.23 🡪 NO.42 |
| 0.057909 | NO.31 🡪 NO.23 🡪 NO.41 | 0.095359 | NO.12 🡪 NO.42 🡪 NO.23 🡪 NO.42 |
| 0.053485 | NO.16 🡪 NO.23 🡪 NO.41 | 0.095342 | NO.7 🡪 NO.42 |
| 0.051129 | NO.41 🡪 NO.12 🡪 NO.23 🡪 NO.41 | 0.092216 | NO.27 🡪 NO.23 🡪 NO.42 |
| 0.049047 | NO.42 🡪 NO.14 🡪 NO.41 | 0.091305 | NO.25 🡪 NO.23 🡪 NO.42 |
| 0.048018 | NO.32 🡪 NO.41 | 0.089992 | NO.16 🡪 NO.23 🡪 NO.42 |
| 0.047741 | NO.42 🡪 NO.12 🡪 NO.23 🡪 NO.41 | 0.077529 | NO.14 🡪 NO.42 🡪 NO.42 |
| 0.047138 | NO.19 🡪 NO.42 🡪 NO.41 | 0.076503 | NO.41 🡪 NO.42 |
| 0.046681 | NO.14 🡪 NO.42 🡪 NO.41 | 0.075577 | NO.41 🡪 NO.42 🡪 NO.42 |
| 0.044705 | NO.1 🡪 NO.23 🡪 NO.41 | 0.074912 | NO.28 🡪 NO.42 🡪 NO.23 🡪 NO.42 |
| 0.041902 | NO.41 🡪 NO.32 🡪 NO.23 🡪 NO.41 | 0.072918 | NO.42 🡪 NO.7 🡪 NO.23 🡪 NO.42 |
| 0.041833 | NO.12 🡪 NO.42 🡪 NO.23 🡪 NO.41 | 0.072339 | NO.30 🡪 NO.23 🡪 NO.42 |
| 0.041286 | NO.28 🡪 NO.42 🡪 NO.41 | 0.070951 | NO.27 🡪 NO.42 🡪 NO.42 |
| 0.039714 | NO.32 🡪 NO.42 🡪 NO.41 | 0.070064 | NO.19 🡪 NO.23 🡪 NO.42 |
| 0.034919 | NO.23 🡪 NO.42 🡪 NO.41 | 0.069069 | NO.30 🡪 NO.42 🡪 NO.42 |
| 0.033369 | NO.15 🡪 NO.14 🡪 NO.41 | 0.06645 | NO.32 🡪 NO.42 |
| 0.032563 | NO.27 🡪 NO.23 🡪 NO.41 | 0.06287 | NO.42 🡪 NO.1 🡪 NO.42 |
| 0.032502 | NO.10 🡪 NO.42 🡪 NO.41 | 0.060867 | NO.26 🡪 NO.13 🡪 NO.42 |
| 0.032322 | NO.10 🡪 NO.41 | 0.059108 | NO.42 🡪 NO.42 🡪 NO.42 🡪 NO.42 |
| 0.031747 | NO.19 🡪 NO.14 🡪 NO.41 | 0.058828 | NO.42 🡪 NO.32 🡪 NO.23 🡪 NO.42 |
| 0.029442 | NO.42 🡪 NO.7 🡪 NO.23 🡪 NO.41 | 0.058624 | NO.7 🡪 NO.42 🡪 NO.42 |
| 0.029239 | NO.25 🡪 NO.23 🡪 NO.41 | 0.058324 | NO.23 🡪 NO.42 🡪 NO.23 🡪 NO.42 |
| 0.028234 | NO.15 🡪 NO.42 🡪 NO.41 | 0.057463 | NO.41 🡪 NO.23 🡪 NO.23 🡪 NO.42 |
| 0.027333 | NO.10 🡪 NO.23 🡪 NO.23 🡪 NO.41 | 0.056451 | NO.15 🡪 NO.14 🡪 NO.42 |
| 0.025847 | NO.14 🡪 NO.23 🡪 NO.23 🡪 NO.41 | 0.055943 | NO.28 🡪 NO.23 🡪 NO.23 🡪 NO.42 |
| 0.025385 | NO.42 🡪 NO.1 🡪 NO.41 | 0.054958 | NO.32 🡪 NO.42 🡪 NO.42 |
| 0.025228 | NO.13 🡪 NO.23 🡪 NO.23 🡪 NO.41 | 0.054398 | NO.8 🡪 NO.23 🡪 NO.42 |
| 0.025054 | NO.27 🡪 NO.42 🡪 NO.41 | 0.052563 | NO.1 🡪 NO.23 🡪 NO.23 🡪 NO.42 |
| 0.024436 | NO.19 🡪 NO.42 🡪 NO.23 🡪 NO.41 | 0.050558 | NO.10 🡪 NO.42 🡪 NO.42 |
| 0.024363 | NO.30 🡪 NO.23 🡪 NO.41 | 0.050278 | NO.10 🡪 NO.42 |
| 0.024199 | NO.14 🡪 NO.42 🡪 NO.23 🡪 NO.41 | 0.047764 | NO.15 🡪 NO.42 🡪 NO.42 |
| 0.023866 | NO.42 🡪 NO.42 🡪 NO.42 🡪 NO.41 | 0.047555 | NO.42 🡪 NO.11 🡪 NO.42 |
| 0.023753 | NO.42 🡪 NO.32 🡪 NO.23 🡪 NO.41 | 0.047387 | NO.12 🡪 NO.28 🡪 NO.42 |
| 0.02354 | NO.29 🡪 NO.23 🡪 NO.23 🡪 NO.41 | 0.046993 | NO.12 🡪 NO.12 🡪 NO.42 |
| 0.023524 | NO.41 🡪 NO.42 🡪 NO.42 🡪 NO.41 | 0.046532 | NO.28 🡪 NO.11 🡪 NO.42 |
| 0.023472 | NO.41 🡪 NO.41 🡪 NO.41 | 0.046019 | NO.18 🡪 NO.23 🡪 NO.42 |
| 0.023262 | NO.30 🡪 NO.42 🡪 NO.41 | 0.045218 | NO.13 🡪 NO.23 🡪 NO.23 🡪 NO.42 |
| 0.023188 | NO.41 🡪 NO.41 🡪 NO.42 🡪 NO.41 | 0.044872 | NO.9 🡪 NO.23 🡪 NO.42 |
| 0.02167 | NO.17 🡪 NO.23 🡪 NO.41 | 0.044808 | NO.18 🡪 NO.42 🡪 NO.42 |
| 0.021402 | NO.28 🡪 NO.42 🡪 NO.23 🡪 NO.41 | 0.044616 | NO.36 🡪 NO.23 🡪 NO.42 |
| 0.020883 | NO.26 🡪 NO.13 🡪 NO.41 | 0.043622 | NO.41 🡪 NO.13 🡪 NO.42 |
| 0.020804 | NO.7 🡪 NO.23 🡪 NO.23 🡪 NO.41 | 0.043506 | NO.27 🡪 NO.28 🡪 NO.42 |
| 0.020789 | NO.15 🡪 NO.23 🡪 NO.23 🡪 NO.41 | 0.043454 | NO.5 🡪 NO.23 🡪 NO.42 |
| 0.020788 | NO.12 🡪 NO.28 🡪 NO.41 | 0.043416 | NO.19 🡪 NO.42 🡪 NO.42 |
| 0.020717 | NO.13 🡪 NO.42 🡪 NO.41 | 0.04308 | NO.28 🡪 NO.28 🡪 NO.28 🡪 NO.42 |
| 0.020616 | NO.12 🡪 NO.12 🡪 NO.41 | 0.042927 | NO.14 🡪 NO.23 🡪 NO.23 🡪 NO.42 |
| 0.020587 | NO.32 🡪 NO.42 🡪 NO.23 🡪 NO.41 | 0.042518 | NO.10 🡪 NO.23 🡪 NO.23 🡪 NO.42 |
| 0.019682 | NO.15 🡪 NO.41 | 0.041308 | NO.12 🡪 NO.12 🡪 NO.42 🡪 NO.42 |
| 0.019201 | NO.42 🡪 NO.11 🡪 NO.41 | 0.041257 | NO.11 🡪 NO.42 🡪 NO.42 |
| 0.01914 | NO.16 🡪 NO.42 🡪 NO.41 | 0.04019 | NO.14 🡪 NO.42 🡪 NO.23 🡪 NO.42 |
| 0.019091 | NO.29 🡪 NO.1 🡪 NO.41 | 0.039178 | NO.41 🡪 NO.42 🡪 NO.23 🡪 NO.42 |
| 0.018826 | NO.7 🡪 NO.7 🡪 NO.23 🡪 NO.41 | 0.038096 | NO.11 🡪 NO.23 🡪 NO.42 |
| 0.018121 | NO.12 🡪 NO.12 🡪 NO.42 🡪 NO.41 | 0.037997 | NO.28 🡪 NO.28 🡪 NO.23 🡪 NO.42 |
| 0.018102 | NO.23 🡪 NO.42 🡪 NO.23 🡪 NO.41 | 0.037138 | NO.31 🡪 NO.23 🡪 NO.23 🡪 NO.42 |
| 0.016849 | NO.10 🡪 NO.42 🡪 NO.23 🡪 NO.41 | 0.037133 | NO.13 🡪 NO.42 🡪 NO.42 |
| 0.01683 | NO.10 🡪 NO.10 🡪 NO.23 🡪 NO.41 | 0.037006 | NO.27 🡪 NO.32 🡪 NO.23 🡪 NO.42 |
| 0.016541 | NO.29 🡪 NO.42 🡪 NO.41 | 0.03678 | NO.27 🡪 NO.42 🡪 NO.23 🡪 NO.42 |
| 0.016232 | NO.19 🡪 NO.23 🡪 NO.23 🡪 NO.41 | 0.035804 | NO.30 🡪 NO.42 🡪 NO.23 🡪 NO.42 |
