# Supplementary material for: Mitigating CO2 emissions associated with digital economy sectors through whole supply chain management
Source: PLoS One. 2025 May 20;20(5):e0323350. doi: 10.1371/journal.pone.0323350 (PMC12091893; doi:10.1371/journal.pone.0323350)
Supplement: S6 Table — (DOCX) [file pone.0323350.s006.docx]

# Supplementary data for:

# Mitigating CO_2_ emissions associated with digital economy sectors through whole supply chain management

**Table S6. The top 100 CO_2_ emission downstream supply chains in the digital economy sectors.**

| Emissions (Mt) | Paths | Emissions (Mt) | Paths |
| --- | --- | --- | --- |
| 0.901 | NO.41 🡪 NO.42 | 12.979 | NO.42 🡪 NO.23 |
| 0.292 | NO.41 🡪 NO.23 | 5.181 | NO.42 🡪 NO.42 |
| 0.252 | NO.41 🡪 NO.42 🡪 NO.23 | 2.770 | NO.42 🡪 NO.23 🡪 NO.23 |
| 0.189 | NO.41 🡪 NO.13 | 2.436 | NO.42 🡪 NO.13 |
| 0.177 | NO.41 🡪 NO.41 | 1.608 | NO.42 🡪 NO.28 |
| 0.101 | NO.41 🡪 NO.42 🡪 NO.42 | 1.451 | NO.42 🡪 NO.42 🡪 NO.23 |
| 0.099 | NO.41 🡪 NO.41 🡪 NO.42 | 0.933 | NO.42 🡪 NO.14 |
| 0.097 | NO.41 🡪 NO.28 | 0.591 | NO.42 🡪 NO.23 🡪 NO.23 🡪 NO.23 |
| 0.062 | NO.41 🡪 NO.23 🡪 NO.23 | 0.579 | NO.42 🡪 NO.42 🡪 NO.42 |
| 0.060 | NO.41 🡪 NO.12 | 0.403 | NO.42 🡪 NO.11 |
| 0.056 | NO.41 🡪 NO.12 🡪 NO.42 | 0.310 | NO.42 🡪 NO.42 🡪 NO.23 🡪 NO.23 |
| 0.054 | NO.41 🡪 NO.42 🡪 NO.23 🡪 NO.23 | 0.298 | NO.42 🡪 NO.26 |
| 0.047 | NO.41 🡪 NO.42 🡪 NO.13 | 0.272 | NO.42 🡪 NO.42 🡪 NO.13 |
| 0.046 | NO.41 🡪 NO.1 | 0.264 | NO.42 🡪 NO.1 |
| 0.040 | NO.41 🡪 NO.14 | 0.233 | NO.42 🡪 NO.28 🡪 NO.28 |
| 0.034 | NO.41 🡪 NO.27 | 0.215 | NO.42 🡪 NO.12 |
| 0.033 | NO.41 🡪 NO.26 | 0.212 | NO.42 🡪 NO.13 🡪 NO.13 |
| 0.032 | NO.41 🡪 NO.41 🡪 NO.23 | 0.202 | NO.42 🡪 NO.12 🡪 NO.42 |
| 0.031 | NO.41 🡪 NO.42 🡪 NO.28 | 0.180 | NO.42 🡪 NO.42 🡪 NO.28 |
| 0.030 | NO.41 🡪 NO.30 🡪 NO.23 | 0.162 | NO.42 🡪 NO.42 🡪 NO.42 🡪 NO.23 |
| 0.028 | NO.41 🡪 NO.42 🡪 NO.42 🡪 NO.23 | 0.162 | NO.42 🡪 NO.27 |
| 0.028 | NO.41 🡪 NO.41 🡪 NO.42 🡪 NO.23 | 0.158 | NO.42 🡪 NO.28 🡪 NO.42 |
| 0.023 | NO.41 🡪 NO.11 | 0.156 | NO.42 🡪 NO.7 |
| 0.021 | NO.41 🡪 NO.7 | 0.148 | NO.42 🡪 NO.41 |
| 0.021 | NO.41 🡪 NO.41 🡪 NO.13 | 0.123 | NO.42 🡪 NO.23 🡪 NO.42 |
| 0.021 | NO.41 🡪 NO.19 🡪 NO.42 | 0.121 | NO.42 🡪 NO.30 🡪 NO.23 |
| 0.020 | NO.41 🡪 NO.41 🡪 NO.41 | 0.118 | NO.42 🡪 NO.14 🡪 NO.14 |
| 0.019 | NO.41 🡪 NO.30 🡪 NO.42 | 0.104 | NO.42 🡪 NO.42 🡪 NO.14 |
| 0.018 | NO.41 🡪 NO.42 🡪 NO.14 | 0.098 | NO.42 🡪 NO.28 🡪 NO.23 |
| 0.016 | NO.41 🡪 NO.13 🡪 NO.13 | 0.085 | NO.42 🡪 NO.14 🡪 NO.42 |
| 0.016 | NO.41 🡪 NO.27 🡪 NO.42 | 0.083 | NO.42 🡪 NO.41 🡪 NO.42 |
| 0.016 | NO.41 🡪 NO.12 🡪 NO.42 🡪 NO.23 | 0.079 | NO.42 🡪 NO.28 🡪 NO.13 |
| 0.015 | NO.41 🡪 NO.16 | 0.078 | NO.42 🡪 NO.27 🡪 NO.42 |
| 0.014 | NO.41 🡪 NO.10 | 0.076 | NO.42 🡪 NO.30 🡪 NO.42 |
| 0.014 | NO.41 🡪 NO.28 🡪 NO.28 | 0.075 | NO.42 🡪 NO.5 🡪 NO.13 |
| 0.014 | NO.41 🡪 NO.12 🡪 NO.13 | 0.071 | NO.42 🡪 NO.23 🡪 NO.13 |
| 0.013 | NO.41 🡪 NO.12 🡪 NO.12 | 0.070 | NO.42 🡪 NO.32 |
| 0.013 | NO.41 🡪 NO.23 🡪 NO.23 🡪 NO.23 | 0.065 | NO.42 🡪 NO.42 🡪 NO.42 🡪 NO.42 |
| 0.013 | NO.41 🡪 NO.19 | 0.064 | NO.42 🡪 NO.7 🡪 NO.42 |
| 0.013 | NO.41 🡪 NO.10 🡪 NO.42 | 0.062 | NO.42 🡪 NO.10 |
| 0.013 | NO.41 🡪 NO.12 🡪 NO.12 🡪 NO.42 | 0.060 | NO.42 🡪 NO.32 🡪 NO.42 |
| 0.012 | NO.41 🡪 NO.27 🡪 NO.13 | 0.056 | NO.42 🡪 NO.12 🡪 NO.42 🡪 NO.23 |
| 0.011 | NO.41 🡪 NO.42 🡪 NO.42 🡪 NO.42 | 0.056 | NO.42 🡪 NO.16 |
| 0.011 | NO.41 🡪 NO.41 🡪 NO.42 🡪 NO.42 | 0.055 | NO.42 🡪 NO.10 🡪 NO.42 |
| 0.011 | NO.41 🡪 NO.41 🡪 NO.41 🡪 NO.42 | 0.055 | NO.42 🡪 NO.27 🡪 NO.13 |
| 0.011 | NO.41 🡪 NO.41 🡪 NO.28 | 0.052 | NO.42 🡪 NO.15 🡪 NO.42 |
| 0.010 | NO.41 🡪 NO.19 🡪 NO.23 | 0.050 | NO.42 🡪 NO.11 🡪 NO.28 |
| 0.010 | NO.41 🡪 NO.28 🡪 NO.42 | 0.050 | NO.42 🡪 NO.12 🡪 NO.13 |
| 0.009 | NO.41 🡪 NO.30 🡪 NO.28 | 0.049 | NO.42 🡪 NO.18 🡪 NO.42 |
| 0.009 | NO.41 🡪 NO.16 🡪 NO.42 | 0.048 | NO.42 🡪 NO.12 🡪 NO.12 |
| 0.009 | NO.41 🡪 NO.7 🡪 NO.42 | 0.048 | NO.42 🡪 NO.18 |
| 0.008 | NO.41 🡪 NO.32 | 0.048 | NO.42 🡪 NO.19 🡪 NO.42 |
| 0.008 | NO.41 🡪 NO.42 🡪 NO.11 | 0.045 | NO.42 🡪 NO.12 🡪 NO.12 🡪 NO.42 |
| 0.007 | NO.41 🡪 NO.32 🡪 NO.42 | 0.045 | NO.42 🡪 NO.11 🡪 NO.42 |
| 0.007 | NO.41 🡪 NO.41 🡪 NO.23 🡪 NO.23 | 0.045 | NO.42 🡪 NO.42 🡪 NO.11 |
| 0.007 | NO.41 🡪 NO.41 🡪 NO.12 | 0.044 | NO.42 🡪 NO.28 🡪 NO.42 🡪 NO.23 |
| 0.006 | NO.41 🡪 NO.30 🡪 NO.23 🡪 NO.23 | 0.042 | NO.42 🡪 NO.15 |
| 0.006 | NO.41 🡪 NO.1 🡪 NO.42 | 0.041 | NO.42 🡪 NO.13 🡪 NO.42 |
| 0.006 | NO.41 🡪 NO.12 🡪 NO.42 🡪 NO.42 | 0.038 | NO.42 🡪 NO.30 🡪 NO.28 |
| 0.006 | NO.41 🡪 NO.41 🡪 NO.12 🡪 NO.42 | 0.037 | NO.42 🡪 NO.8 🡪 NO.42 |
| 0.006 | NO.41 🡪 NO.16 🡪 NO.23 | 0.036 | NO.42 🡪 NO.11 🡪 NO.23 |
| 0.006 | NO.41 🡪 NO.28 🡪 NO.23 | 0.036 | NO.42 🡪 NO.1 🡪 NO.42 |
| 0.006 | NO.41 🡪 NO.5 🡪 NO.13 | 0.035 | NO.42 🡪 NO.16 🡪 NO.42 |
| 0.006 | NO.41 🡪 NO.12 🡪 NO.1 | 0.035 | NO.42 🡪 NO.23 🡪 NO.42 🡪 NO.23 |
| 0.006 | NO.41 🡪 NO.42 🡪 NO.26 | 0.034 | NO.42 🡪 NO.28 🡪 NO.28 🡪 NO.28 |
| 0.006 | NO.41 🡪 NO.19 🡪 NO.42 🡪 NO.23 | 0.033 | NO.42 🡪 NO.42 🡪 NO.26 |
| 0.005 | NO.41 🡪 NO.20 🡪 NO.42 | 0.032 | NO.42 🡪 NO.8 |
| 0.005 | NO.41 🡪 NO.42 🡪 NO.42 🡪 NO.13 | 0.031 | NO.42 🡪 NO.11 🡪 NO.13 |
| 0.005 | NO.41 🡪 NO.30 🡪 NO.42 🡪 NO.23 | 0.030 | NO.42 🡪 NO.42 🡪 NO.42 🡪 NO.13 |
| 0.005 | NO.41 🡪 NO.41 🡪 NO.42 🡪 NO.13 | 0.030 | NO.42 🡪 NO.19 |
| 0.005 | NO.41 🡪 NO.42 🡪 NO.1 | 0.030 | NO.42 🡪 NO.7 🡪 NO.7 |
| 0.005 | NO.41 🡪 NO.14 🡪 NO.14 | 0.030 | NO.42 🡪 NO.42 🡪 NO.1 |
| 0.005 | NO.41 🡪 NO.41 🡪 NO.1 | 0.029 | NO.42 🡪 NO.6 |
| 0.005 | NO.41 🡪 NO.15 🡪 NO.42 | 0.028 | NO.42 🡪 NO.29 🡪 NO.42 |
| 0.005 | NO.41 🡪 NO.18 🡪 NO.42 | 0.027 | NO.42 🡪 NO.41 🡪 NO.23 |
| 0.005 | NO.41 🡪 NO.34 🡪 NO.42 | 0.026 | NO.42 🡪 NO.23 🡪 NO.23 🡪 NO.42 |
| 0.005 | NO.41 🡪 NO.18 | 0.026 | NO.42 🡪 NO.42 🡪 NO.28 🡪 NO.28 |
| 0.005 | NO.41 🡪 NO.28 🡪 NO.13 | 0.026 | NO.42 🡪 NO.6 🡪 NO.42 |
| 0.005 | NO.41 🡪 NO.8 🡪 NO.42 | 0.026 | NO.42 🡪 NO.30 🡪 NO.23 🡪 NO.23 |
| 0.005 | NO.41 🡪 NO.27 🡪 NO.42 🡪 NO.23 | 0.025 | NO.42 🡪 NO.22 🡪 NO.23 |
| 0.005 | NO.41 🡪 NO.42 🡪 NO.28 🡪 NO.28 | 0.024 | NO.42 🡪 NO.42 🡪 NO.12 |
| 0.004 | NO.41 🡪 NO.41 🡪 NO.14 | 0.024 | NO.42 🡪 NO.14 🡪 NO.42 🡪 NO.23 |
| 0.004 | NO.41 🡪 NO.42 🡪 NO.12 | 0.024 | NO.42 🡪 NO.42 🡪 NO.13 🡪 NO.13 |
| 0.004 | NO.41 🡪 NO.8 | 0.024 | NO.42 🡪 NO.16 🡪 NO.23 |
| 0.004 | NO.41 🡪 NO.42 🡪 NO.13 🡪 NO.13 | 0.023 | NO.42 🡪 NO.41 🡪 NO.42 🡪 NO.23 |
| 0.004 | NO.41 🡪 NO.7 🡪 NO.7 | 0.023 | NO.42 🡪 NO.28 🡪 NO.28 🡪 NO.42 |
| 0.004 | NO.41 🡪 NO.15 | 0.023 | NO.42 🡪 NO.12 🡪 NO.42 🡪 NO.42 |
| 0.004 | NO.41 🡪 NO.27 🡪 NO.14 | 0.023 | NO.42 🡪 NO.42 🡪 NO.12 🡪 NO.42 |
| 0.004 | NO.41 🡪 NO.42 🡪 NO.12 🡪 NO.42 | 0.022 | NO.42 🡪 NO.19 🡪 NO.23 |
| 0.004 | NO.41 🡪 NO.12 🡪 NO.7 | 0.022 | NO.42 🡪 NO.31 🡪 NO.42 |
| 0.004 | NO.41 🡪 NO.41 🡪 NO.27 | 0.022 | NO.42 🡪 NO.27 🡪 NO.42 🡪 NO.23 |
| 0.004 | NO.41 🡪 NO.12 🡪 NO.23 | 0.021 | NO.42 🡪 NO.12 🡪 NO.1 |
| 0.004 | NO.41 🡪 NO.31 🡪 NO.42 | 0.021 | NO.42 🡪 NO.30 🡪 NO.42 🡪 NO.23 |
| 0.004 | NO.41 🡪 NO.14 🡪 NO.42 | 0.021 | NO.42 🡪 NO.13 🡪 NO.26 |
| 0.004 | NO.41 🡪 NO.41 🡪 NO.26 | 0.021 | NO.42 🡪 NO.28 🡪 NO.23 🡪 NO.23 |
| 0.004 | NO.41 🡪 NO.10 🡪 NO.42 🡪 NO.23 | 0.020 | NO.42 🡪 NO.11 🡪 NO.11 |
| 0.004 | NO.41 🡪 NO.41 🡪 NO.41 🡪 NO.23 | 0.020 | NO.42 🡪 NO.42 🡪 NO.42 🡪 NO.28 |
| 0.003 | NO.41 🡪 NO.42 🡪 NO.42 🡪 NO.28 | 0.020 | NO.42 🡪 NO.9 🡪 NO.42 |
| 0.003 | NO.41 🡪 NO.41 🡪 NO.42 🡪 NO.28 | 0.019 | NO.42 🡪 NO.24 🡪 NO.28 |
| 0.003 | NO.41 🡪 NO.41 🡪 NO.30 🡪 NO.23 | 0.019 | NO.42 🡪 NO.27 🡪 NO.14 |
